# Complete genome sequence of *Bacillus stercoris* BST19, a soil isolate with potential anti-microbial properties using PacBio sequencing

**DOI:** 10.1128/mra.00396-24

**Published:** 2024-08-23

**Authors:** Hsin-Tzu Ku, Marcia Shu-Wei Su, Yu-Chia Yeh, Shane Marie Coronel, Chi-Chung Chen, Hung-Yu Shu

**Affiliations:** 1 Department of Bioscience Technology, Chang Jung Christian University, Tainan, Taiwan; 2 Department of Medical Laboratory Science and Biotechnology, College of Medical and Health Sciences, Asia University, Taichung, Taiwan; 3 School of Chemical, Biological, and Materials Engineering and Sciences, Mapua University, Manila, Philippines; 4 Department of Medical Research, Chi Mei Medical Center, Tainan, Taiwan; University of Strathclyde, Glasgow, United Kingdom

**Keywords:** *Bacillus stercoris*, soil isolate, PacBio sequencing

## Abstract

We report the complete genome sequence of *Bacillus stercoris* BST19, an isolate from the allotment soil in Tainan, Taiwan. The genome was obtained using the PacBio Sequel II platform, yielding a circular chromosome of 4,167,147 bp with a 43.9% GC content.

## ANNOUNCEMENT

Bioactive secondary metabolite-producing *Bacillus* species have reportedly been studied for beneficial applications in agricultural bio-controls, environmental waste degradation, and biomedical treatments (1–3). Due to the great potential of the antimicrobial properties that naturally occurring *Bacillus* may have, we sequenced a soil isolate *B. stercoris* BST19.

*B. stercoris* BST19 was isolated from cultivated soil (four-inch depth) of allotments located in Tainan, Taiwan (22.9036° N, 120.2722° E). The soil sample (two grams) was homogenized, diluted in phosphate buffer saline without filtration, and plated on nutrient broth (NB) agar for incubation at 37°C for 24 h. To ensure a pure culture, a colony was re-streaked twice on NB agar and cultured in NB medium at 37°C for 18 h for genomic DNA (gDNA) isolation. We extracted gDNA from the bacterial pellets (centrifugation: 15,000 *g*, 2 min) using the NucleoBond AXG 20 kit (Macherey-Nagel, Düren, Germany). The high integrity gDNA samples were then sheared into 10–15 kb fragments using the g-TUBE (Covaris, MA, USA), and purified with AMPurePB beads of Pacific Biosciences (PacBio, CA, USA). We used the SMRTbell express template prep kit 2.0 (PacBio) to construct our library, followed by a size selection (cutoff: 10 kb) using BluePippin (Sage Science, USA). Next, the library was sequenced on a SMRT cell (the PacBio Sequel IIe platform, 2.0 chemistry) that produced 1,778,504 polymerase reads and 4,473,417 subreads (*N_50_* = 8,035) with 6,194 × coverage. The raw data were run through a continuous long reads (CLR) sequencing application (PacBio) to generate reads. The CLR reads exceeding 1 kb were acquired by SeqKit (v.2.3.0, PacBio) and then *de novo* assembled using Hierarchical Genome Assembly Process pipeline (v.4.0) with default parameters integrated into the PacBio SMRT Link (v.10.1.0). We used BLASTn (BLAST +2.15.0) to identify two overlapping regions (8,833 and 7,492 bases) at the ends of the replicon and trimmed, allowing us to obtain one assembled circular chromosome measuring 4,167,147 bp with a 43.9% GC content. The trimmed sequence without rotating was annotated using the DDBJ Fast Annotation and Submission Tool (v.1.6.0) (4), and predicted to have 4,093 coding sequences, 86 tRNA genes, and 10 rRNA operons.

We classified our *B. stercoris* strain BST19 using the Type (Strain) Genome Server (v.391) (5) for genome-based prokaryote taxonomy. This analysis calculated the digital DNA–DNA hybridization (dDDH) values among *B. stercoris* BST19 and *Bacillus* species, with the highest dDDH value (88.3%) to *B. stercoris* strain D7XPN1 (genome assembly accession: GCA_000738015). To characterize biosynthetic gene clusters in the BST19 genome, we executed genome mining using antiSMASH (v.6.0) (6). Our analysis predicted 14 gene clusters encoding for non-ribosomal peptide synthetases (NRPS), polyketides synthases (PKS), and hybrid NRPS/PKS. These enzymes presumably produce antimicrobial compounds including fengycin, surfactin, and bacillaene. Collectively, our analyses suggest that *B. stercoris* BST19 may possess antagonistic activity and antimicrobial properties. Unless specified otherwise, default parameters were applied in all the software used.

## Data Availability

The *B. stercoris* BST19 genome sequence has been deposited in the DDBJ/EMBL/GenBank (accession number: NZ_AP029050), BioProject (PRJDB16835), BioSample (SAMD00652217), and Sequence Read Archive (DRR517365).
